# BMP7 alleviates trigeminal neuralgia by reducing oligodendrocyte apoptosis and demyelination

**DOI:** 10.1186/s10194-023-01681-3

**Published:** 2023-10-24

**Authors:** Kai Chen, Xiaojin Wei, Ruixuan Wang, Lin Yang, Dingquan Zou, Yaping Wang

**Affiliations:** 1 Department of Pain Management and Anesthesiology, The Second Xiangya Hospital, Central South University, Changsha, Hunan China; 2 Clinical Research Center for Pain Medicine in Hunan Province, Changsha, Hunan China; 3 School of Basic Medical Sciences, Capital Medical University, Beijing, 100054 China; 4 Bourns Engineering, The University of California, Riverside, CA 92521 USA

**Keywords:** Trigeminal neuralgia, BMP7, Demyelination, Oligodendrocytes, Apoptosis

## Abstract

**Background:**

BMP7 has been shown to have neuroprotective effects and to alleviate demyelination. However, its role in trigeminal neuralgia (TN) has not been well investigated. The current study aims to determine whether BMP7 plays a role in demyelination, its effects on pain behaviors and mechanism of action in rats with TN.

**Methods:**

We used an infraorbital-nerve chronic-constriction injury (ION-CCI) to establish a rat model of TN. Adeno-associated viruses (AAVs) were injected into the rats to upregulate or downregulate BMP7. The mechanical withdrawal thresholds (MWT) of the injured rats were detected using Von Frey filaments. The changes in expression levels of BMP7 and oligodendrocyte (OL) markers were examined by western blotting, quantitative real-time PCR, immunofluorescence, and transmission electron microscopy.

**Results:**

The ION-CCI induced mechanical allodynia, demyelination, and loss of OLs with a reduction of BMP7. Short-hairpin RNA (shRNA)-BMP7 that inhibited BMP7 expression also caused mechanical allodynia, demyelination, and loss of OLs, and its mechanism may be OL apoptosis. Overexpressing BMP7 in the trigeminal spinal subnucleus caudalis(VC) with AAV-BMP7 relieved all three phenotypes induced by the CCI, and its mechanism may be alleviating OLs apoptosis. Two signal pathways associated with apoptosis, STAT3 and p65, were significantly downregulated in the VC after CCI and rescued by BMP7 overexpression.

**Conclusion:**

BMP7 can alleviate TN by reducing OLs apoptosis and subsequent demyelination. The mechanism behind this protection could be BMP7-mediated activation of the STAT3 and NF-κB/p65 signaling pathway and subsequent decrease in OL apoptosis. Importantly, our study presents clear evidence in support of BMP7 as a possible therapeutic target for the treatment of TN.

## Introduction

Trigeminal neuralgia (TN) is a chronic, neuropathic pain characterized by paroxysms of spontaneous and evoked shock-like or stabbing pain in an area of the face [1]. The incidence of trigeminal neuralgia rises with age, and medication is the first choice for the treatment, but quality of life is poor in patients even with optimal drug regimens [2]. Therefore, treating TN is still a medical challenge. Although great progress has been made, specific molecular mechanisms of TN remain unclear. Current studies suggest that the transitionary area where the trigeminal nerve enters the pons is vulnerable to damage, particularly to demyelination. Vascular compression is the usual cause of demyelination at the site just before the nerve enters the pons, and multiple sclerosis is the typical cause at the site just after entry into the pons [1]. When the myelin sheath of the axon becomes thin enough to allow ions to pass through its membrane, the axon loses its ability to pump out sodium in a timely manner [1]. The resulting depolarized resting membrane potential makes the axon hyperexcitable [3], causing ectopic generation of impulses with high-frequency after-discharges [4, 5] (discharges that occur after termination of the stimulus) and cross-talk between fibers (called ephaptic transmission) [6].

Bone morphogenetic protein 7 (BMP7) belongs to the transforming growth factor-β (TGF-β) superfamily and plays a neuroprotective role in many different models of neurological disease. BMP7 has been reported to reduce ischemia-induced injury in the adult cerebral cortex [7], and reduce cerebral ischemia/reperfusion injury by attenuating oxidative stress and inhibiting neuronal apoptosis [8]. Researchers have demonstrated that upregulation of BMP7 greatly increased the number of oligodendrocytes (OLs) in vitro [9] and induced oligodendrogenesis in rats with spinal cord injury (SCI) [10]. Additionally, previous results from our group have shown that BMP7 could profoundly inhibit TNF-α-induced OL apoptosis in vitro [11] and that overexpression of BMP7 could reduce oligodendrocyte loss after SCI [12]. However, whether BMP7 can relieve trigeminal neuralgia by reducing OL loss and subsequent demyelination remains to be fully elucidated.

Currently, there are no studies investigating the analgesic effects of BMP7 by reducing demyelination. The primary purpose of our study is to investigate whether BMP7 can play a role in demyelination and the effect of this on pain behavior in rats with TN. To this effect, we hypothesized that BMP7 alleviates TN by decreasing OL apoptosis and subsequent demyelination. Moreover, we also investigated the STAT3/p65 signaling pathway as a possible mechanism of action of BMP7.

## Materials and methods

### Animals

Adult male Sprague-Dawley rats weighing 200 to 250 g were purchased from Central South University Animal Services and housed separately in a temperature- and humidity-controlled room, with ad libitum food and water and 12/12 hour light/dark cycles. All of the experimental procedures were approved by the Animal Ethics Committee of Central South University and conducted in accordance with the National Institutes of Health Guide for the Care and Use of Laboratory Animals.

### Trigeminal neuralgia model

The TN model was performed using an infraorbital-nerve chronic-constriction injury (ION-CCI) according to the method described by Ding et al. [13]. Briefly, rats were anesthetized with an intraperitoneal (i.p.) injection of pentobarbital (40 mg/kg), and a 0.5 cm incision was made in the facial skin between the left eye and whisker pad to expose the distal segment of the ION. Two ligatures of 4 − 0 chromic catgut were loosely tied around the distal branch of the ION (2 mm apart). To ensure proper constriction of the ION, a criterion proposed by Bennett and Xie was followed [14]. Finally, the facial skin was closed with 4 − 0 polyester suture. Rats in the sham group underwent the same surgical procedure including skin incision and the ION nerve dissection but no nerve ligation.

### Stereotaxic viral injection

**Fig. 1:**
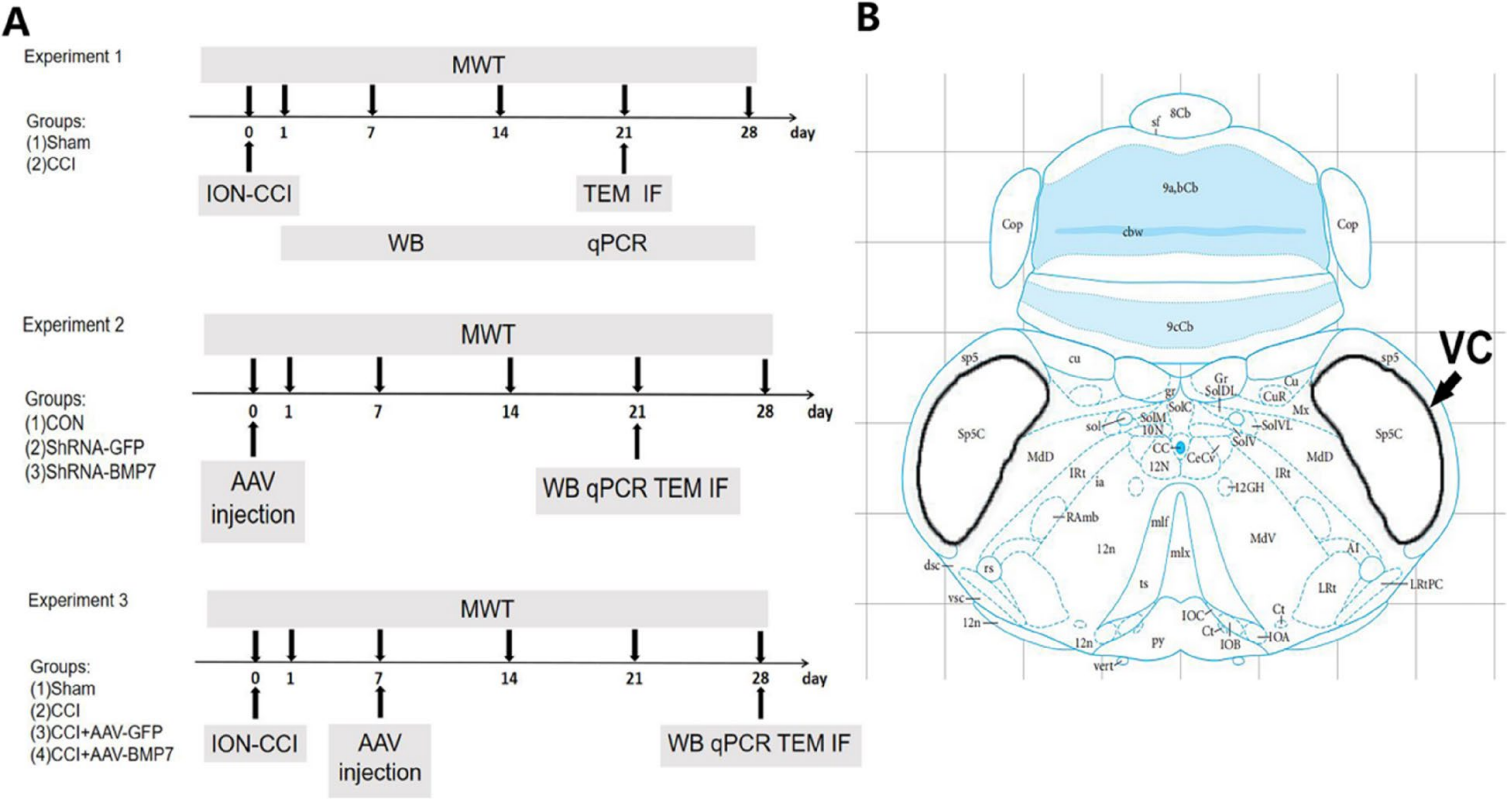
Schematic representation of the experimental designs and the left trigeminal spinal subnucleus caudalis (VC) brain region. **A** Timeline of the experimental designs and animal groups (for details see the “Materials and methods” section in the text). **B** A coronal view of the rat brain shows the location of the VC (The Rat Brain in Stereotaxic Coordinates, Sixth Edition). CCI = chronic constriction injury; ION – infraorbital nerve; MWT = mechanical withdrawal threshold; TEM = transmission electron microscopy; IF = immunofluorescence; WB = western blot; qPCR = real-time quantitative polymerase chain reaction; CON = control; shRNA = short hairpin RNA; AAV = adeno-associated virus

We selected adeno-associated virus (AAV) 9, which is susceptible to transfection into nervous tissue, to overexpress (Braincase Company) or inhibit (Hanbio Biotechnology) BMP7. The concentrations of the AAV-BMP7-GFP vector and its control vector AAV-GFP were 5.1 × 10^12^ vg/ml and 3.2 × 10^12^ vg/ml respectively. These vectors will henceforth be referred to as AAV-BMP7 and AAV-GFP. The concentration of the AAV-shRNA-BMP7-GFP and its control vector AAV-shRNA-Control-GFP was 1.4 × 10^12^ vg/ml and 1.7 × 10^12^ vg/ml respectively. These vectors will henceforth be referred to as shRNA-BMP7 and shRNA-GFP. The rats were deeply anesthetized (i.p. injection of 40 mg/kg pentobarbital) and fixed on a stereotaxic apparatus (RWD Life Science). The left trigeminal spinal subnucleus caudalis (VC) was accurately located according to the stereotaxic atlas (The Rat Brain in Stereotaxic Coordinates, Sixth Edition) at the following stereotaxic coordinates (from bregma): AP: −14.50 mm, ML: −2.40 mm, DV: −8.60 mm [15]. A local microinjection was performed with a 33-gauge Hamilton syringe at a controlled speed of 0.2 µl/min, and 1 µl AAV was injected into the VC. After the injection, the needle of the syringe was held in place for 15 min to prevent backflow and was then slowly retracted. Finally, the wound was closed with (Fig. 1).

### Mechanical withdrawal threshold

Von Frey filaments (Stoelting, IL, USA) were used to test the rats’ mechanical withdrawal threshold (MWT) by a researcher who was unfamiliar with the experimental groups. Animals were allowed to acclimatize to the testing apparatus for one week before beginning the Von Frey test (0.6, 1.0, 1.4, 2.0, 4.0, 6.0, 8.0, 10.0, and 15.0 g filaments). The MWT was measured from pre-operation to 28 days after the surgery. Animals were placed in individual cages, where they were kept for 30 min in order to adapt to the new environment. The Von Frey filaments were applied within the ION region, near the center of the facial whisker pad. Brisk head withdrawals, escape or attack reactions, or short-lasting facial grooming were identified as a positive sign of a withdrawal. The up-down method was used to determine the 50% MWT.

### Western blotting

Rats were deeply anesthetized and then rapidly euthanized. The VC was harvested on ice and kept in radio-immunoprecipitation assay (RIPA) lysis buffer. Samples were separated by 8–15% sodium dodecyl sulfate–polyacrylamide gel electrophoresis (SDS-PAGE), and then transferred to polyvinylidene fluoride (PVDF) membranes. After blocking non-specific proteins with 10% non-fat milk for 1 h, the membranes were incubated overnight at 4 °C with the following primary antibodies: GAPDH (1:10000, Proteintech, 10494-1-AP), BMP7 (1:1000, Bioss, bs2242R), CNPase (1:2000, Abcam, ab6319), MBP (1:5000, Abcam, ab218011), Bax (1:2000, Proteintech, 50599-2-lg), Bcl-2 (1:1000, Proteintech, 26593-1-AP), P-STAT3 (1:2000, CST, 9145), STAT3 (1:1000, CST, 30835), P-P65 (1:1000, CST, 3033), P65 (1:1000, CST, 8242). The following day, the membranes were washed with tris-buffered saline Tween-20 (TBST) and incubated with the appropriate horseradish peroxidase (HRP)-conjugated secondary antibodies for 1 h at 37 °C. After reacting with the chemiluminescent HRP substrate (Millipore), the immunoreactivity bands were photographed using an imaging system (Clinx Science Instrument). The optical densities of the bands were quantified using ImageJ software.

### Quantitative real-time polymerase chain reaction (qPCR)

After the left VC was harvested on ice, total RNA was extracted by Trizol reagent (TIANGEN BIOTECH, DP424) and then reverse-transcribed into complementary DNA (cDNA) using a cDNA Synthesis Kit (TIANGEN BIOTECH, KR118). qPCR was performed using the Real-Time Quantitative PCR SYBR Green kit (Novoprotein, E096) on the PCR System (Bio-Rad Laboratories). The primer sequences were as follows: 

- GAPDH forward:5′-ACCATCTTCCAGGAGCGAGA-3′, reverse: GGCGGAGATGATGACCCT; BMP7 forward:5′-GCCTGGACAACGAGGTG-3′, reverse: AGCCCAAGATGGACAGGA; CNPase forward:5′-TCCGAGGAGTACAAGCGTCT-3′, reverse: ACAGCTGCCATTGGTTCTTC; and MBP forward:5′-GGCATCACAGAAGAGACCCTCAC-3′, reverse: GCCCGATGGAGTCAAGGATG.

### Immunofluorescence

Animals were anesthetized (i.p. injection of 40 mg/kg pentobarbital) before perfusion with a solution of 0.9% sodium chloride and 4% paraformaldehyde (PFA). The medulla oblongata was removed and placed in 4% PFA overnight at 4 °C, and then transferred to 30% sucrose in phosphate-buffered saline (PBS) for cryoprotection. An area outside our region of interest, on the right side of the medulla oblongata was marked to distinguish the left and right of the sections. The tissues were cut into 10-µm sections by a freezing microtome (Leica). The sections were washed in 0.3% Triton X-100 for 10 min and then blocked in 5% bovine serum albumin (BSA) before incubation with primary antibodies overnight at 4 °C. The next day, the sections were incubated with the appropriate secondary antibodies diluted in PBS for 1 h at room temperature. Primary antibodies included the following: BMP7 (1:400, Bioss, bs2242R), APC (1:400, Abcam, ab16794), P-STAT3 (1:200, CST, 9145), P-P65 (1:400, CST, 3033). Secondary antibodies included the following: Cy3-conjugated anti-mouse IgG (1:200, Servicebio, GB21301), FITC conjugated anti-rabbit IgG (1:200, Servicebio, GB22403), Cy5-conjugated anti-mouse IgG (1:200, Servicebio, GB27301), Cy5-conjugated anti-rabbit IgG (1:200, Servicebio, GB27303). Finally, DAPI (Servicebio, G1012) was added to the sections for 10 min. Apoptotic cells were identified using a terminal deoxynucleotidyl transferase-mediated dUTP nick end labeling (TUNEL) Cell-Apoptosis Detection Kit (Servicebio, G1502) according to the manufacturer’s protocol before incubation with primary antibodies. The stained sections were covered and photographed using fluorescence microscopy (OLYMPUS). The field of view to measure TUNEL staining on the VC was randomly selected. The fluorescence intensity and the percentage of co-stain were quantified using ImageJ software.

### Transmission electron microscopy (TEM)

After being deeply anesthetized (i.p. injection of 40 mg/kg pentobarbital), the rats were euthanized, and their brains extracted. One mm^3^ of fresh tissue from the left VC was harvested within 3 min post-euthanasia and put into TEM fixative (Servicebio, G1102) at 4 °C for preservation. The tissue was post-fixed with 1% osmium tetroxide (OsO_4_) dissolved in 0.1 M PBS (pH 7.4) for 2 h at room temperature and dehydrated in gradually ascending concentration series (30–100%) of ethanol. After embedding with EMBed 812 (SPI, 90529-77-4), specimens were kept at 37 °C overnight and then moved to 60 °C to polymerize for more than 48 h before being cut into 60–80 nm sections with the ultramicrotome (Leica). An alcohol solution saturated with 2% uranium acetate and 2.6% lead citrate was used to stain in sequence. Finally, all of the thin sections were observed under a transmission electron microscope (HITACHI). G-ratios of myelinated axons were measured with ImageJ.

### Statistical analysis

All of the data are presented as the mean ± standard deviation (SD). The unpaired Student’s t-test and ANOVA were used to analyze the significance of differences. All data were evaluated using GraphPad Prism. Differences of *p* < 0.05 were considered statistically significant.

## Results

### ION-CCI induced mechanical allodynia and demyelination with a reduction of BMP7

**Fig. 2:**
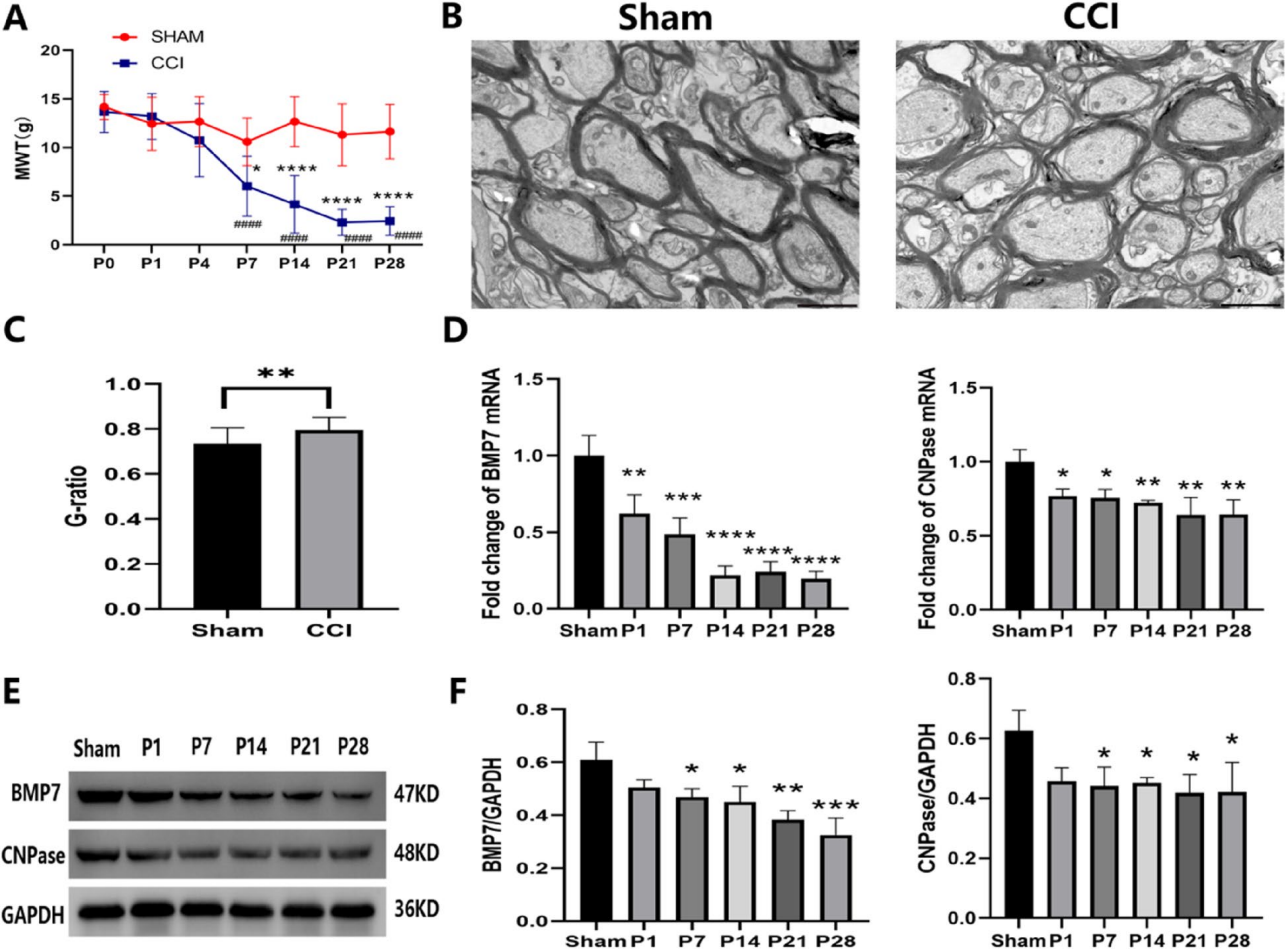
ION-CCI induced mechanical allodynia and demyelination with a reduction of BMP7. **A** MWT of rats in the CCI group and the Sham group at the indicated time points post-operation (P); *n* = 6 per group. **B** TEM images showed the myelin profiles in the VC of Sham and CCI groups at day 21 after ION-CCI; scale bar = 20 μm. **C** G-ratios of myelin with different diameters that were calculated from (**B**); *n* = 3 per group. **D** mRNA levels measured by qPCR of BMP7 and CNPase in the VC of CCI and Sham groups at the corresponding time points post-operation (P); *n* = 3 per group. mRNA quantification is presented as a fold change of CCI/Sham. **E**, **F** Protein levels measured by western blotting of BMP7 and CNPase in the VC of CCI and Sham groups at the corresponding time points post-operation; *n* = 3 per group. The bands are imaged in (**E**) and the quantification is presented in (**F**). GAPDH is used as the loading control. The optical densities of each band are indicated in kiloDaltons (kD). Compared with the Sham group, *p < 0.05, **p < 0.01, ***p < 0.001, ****p < 0.0001. Compared with the baseline (P0), ^####^*p* < 0.0001. MWT = Mechanical withdrawal threshold; ION-CCI = Infraorbital-nerve chronic constriction injury; VC = left trigeminal spinal subnucleus caudalis; TEM = Transmission electron microscopy

**Fig. 3:**
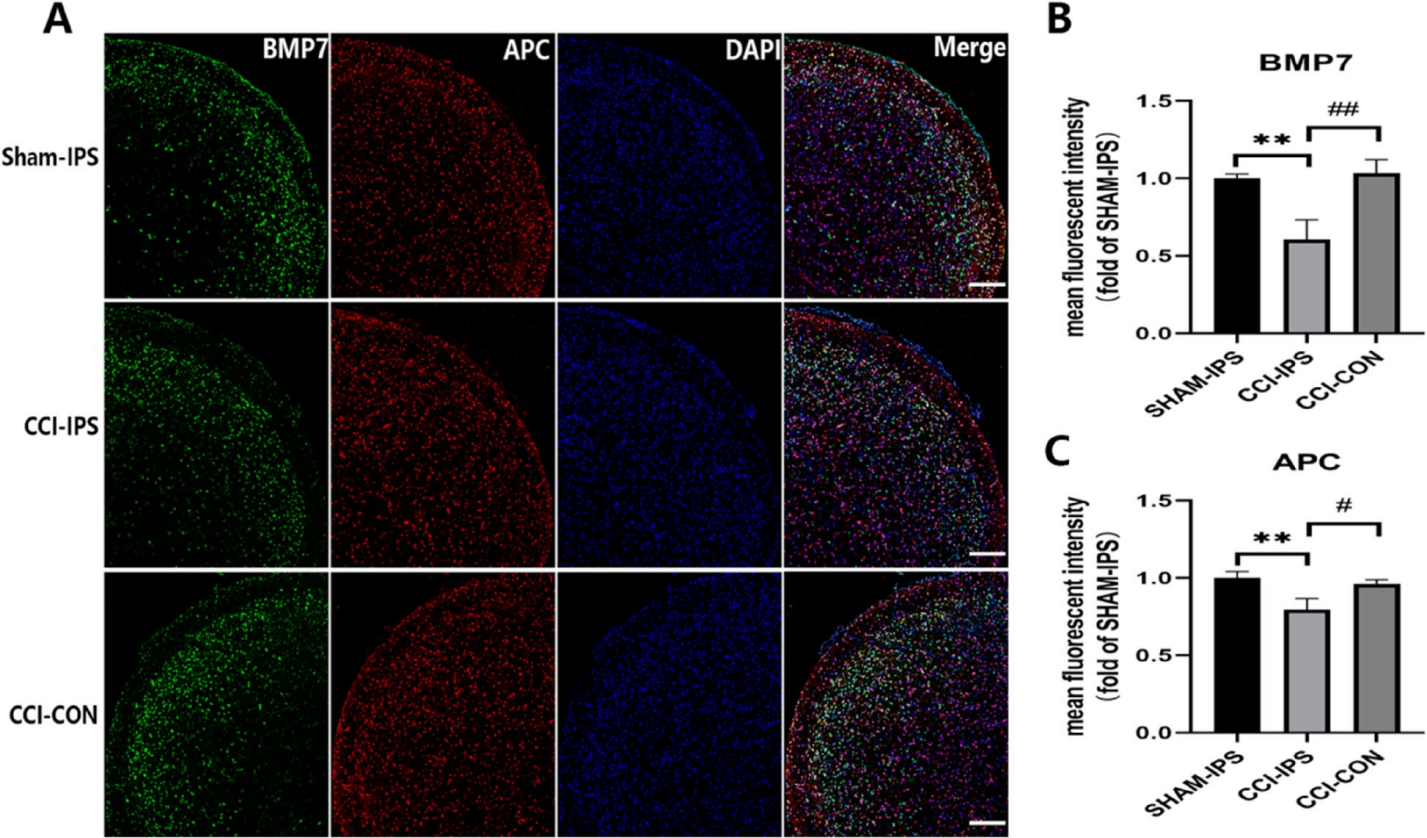
ION-CCI caused the loss of OLs and BMP7. **A** Immunofluorescence staining for BMP7 and APC in the VC ipsilateral (IPS) or contralateral (CON) to the injury in the Sham and CCI groups at day 21 after ION-CCI; scale bar = 200 μm. **B**, **C** Mean fluorescence intensity of BMP7 and APC were calculated from (**A**); *n* = 3 per group. **p < 0.01 compared with the Sham-IPS group, ^#^*p* < 0.05 compared with the CCI-CON group, ^##^*p* < 0.01 compared with the CCI-CON group. ION-CCI = infraorbital-nerve chronic constriction injury; OL = oligodendrocyte; VC = trigeminal spinal subnucleus caudalis

The TN model using ION-CCI induced persistent pain in rats. Compared with the Sham group or baseline (P0), the MWT of the ION-CCI group was significantly reduced in the ipsilateral ION territory beginning at day 7 and persisting till at least day 28 after surgery (Fig. 2A). TEM images showed that some of the myelin sheaths were thin, loose and even fractured in the CCI group, while in Sham group, myelin sheaths were thicker and denser (Fig. 2B). The G-ratio metric, the ratio of the axonal diameter to the total outer diameter of the nerve fiber, revealed that myelin sheaths in the Sham group were thicker and more proportional to the axon diameters (*p* < 0.01, Fig. 2C). The mRNA of CNPase (a marker of OLs) decreased from day 1 (*p* < 0.05) and continued to diminish until day 28 (*p* < 0.01) after CCI. Similarly, we found that the mRNA of BMP7 showed the same expression pattern as CNPase with a clear reduction over time (Fig. 2D). Moreover, the protein levels of both CNPase and BMP7 were also significantly reduced beginning at day 7 and persisting to day 28 after CCI (Fig. 2E–F). As shown in Fig. 3, CCI induced a reduction of ipsilateral BMP7 and APC (a marker of OLs) in VC at day 21 after surgery. In addition, compared with the contralateral VC, CCI also induced loss of ipsilateral BMP7 and APC in VC. These observations motivated us to further explore whether the expression of BMP7 is related to the reduction of OLs after CCI.

### shRNA-BMP7 induced mechanical allodynia and demyelination

**Fig. 4:**
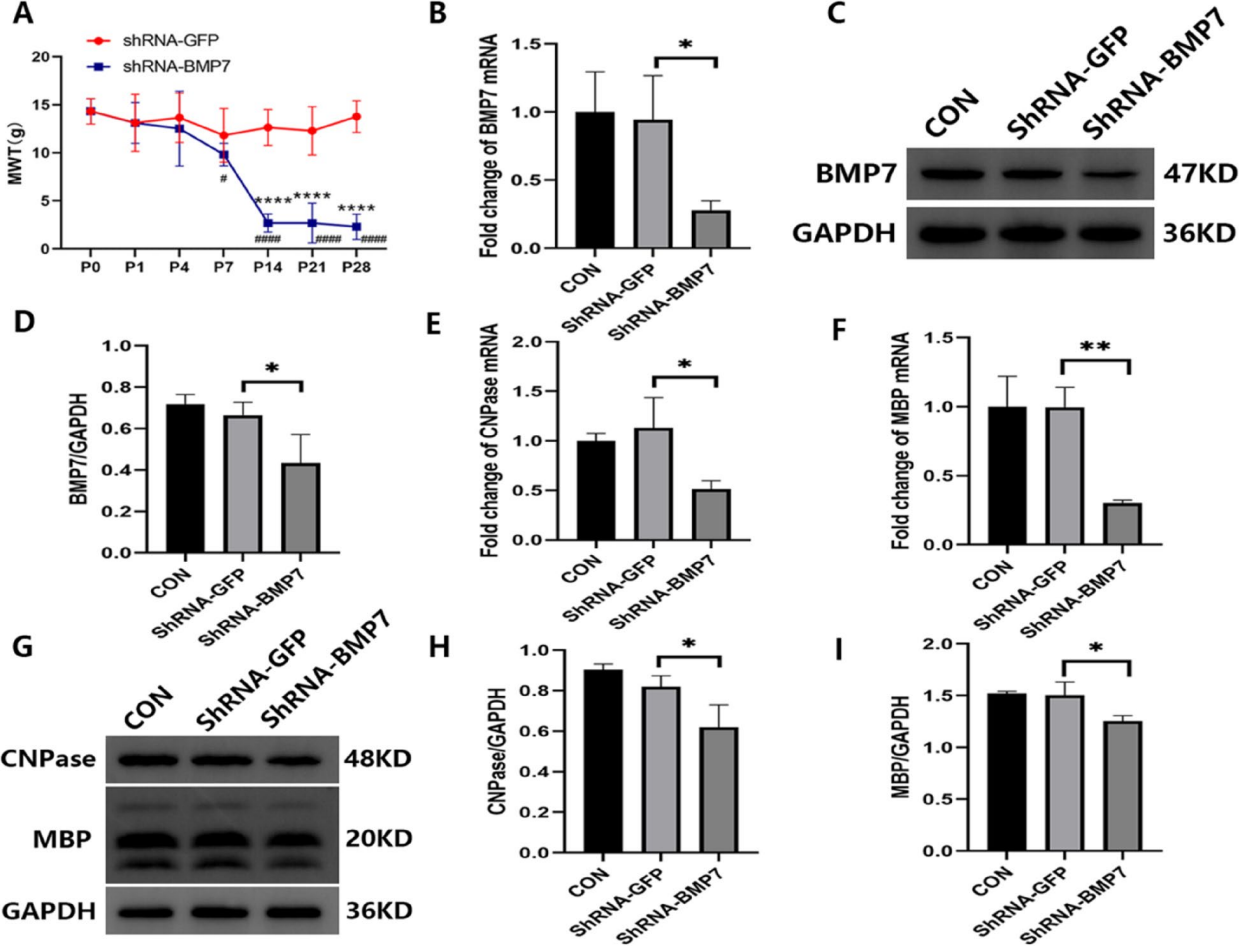
ShRNA-BMP7 induced mechanical allodynia and demyelination. **A** MWT of rats in the shRNA-GFP and shRNA-BMP7 groups at the corresponding time points after the AAV injection; *n* = 6 per group. **B**–**D** mRNA and protein levels of BMP7 in the VC of control group (CON), shRNA-GFP group and shRNA-BMP7 group at day 21 after AAV injection; *n* = 3 per group. **E**–**I** mRNA and protein levels of OL markers (CNPase and MBP) in the VC of CON, shRNA-GFP, and shRNA-BMP7 groups at day 21 after AAV injection; *n* = 3 per group. mRNA levels in (**B**), (**E**), and (**F**) are presented as a fold change from the CON group mRNA level. Protein bands depicted in (**C**) and (**G**) have the corresponding optical densities labeled in kiloDaltons (kD). Quantifications of these bands are shown in (**D**), (**H**), and (**I**), normalized by the GAPDH loading control. Compared with the shRNA-GFP, *p < 0.05, **p < 0.01, ****p < 0.0001. Compared with the baseline (P0), ^#^*p* < 0.05, ^####^*p* < 0.0001. shRNA = short hairpin RNA; VC = trigeminal spinal subnucleus caudalis; AAV = adeno-associated virus

**Fig. 5:**
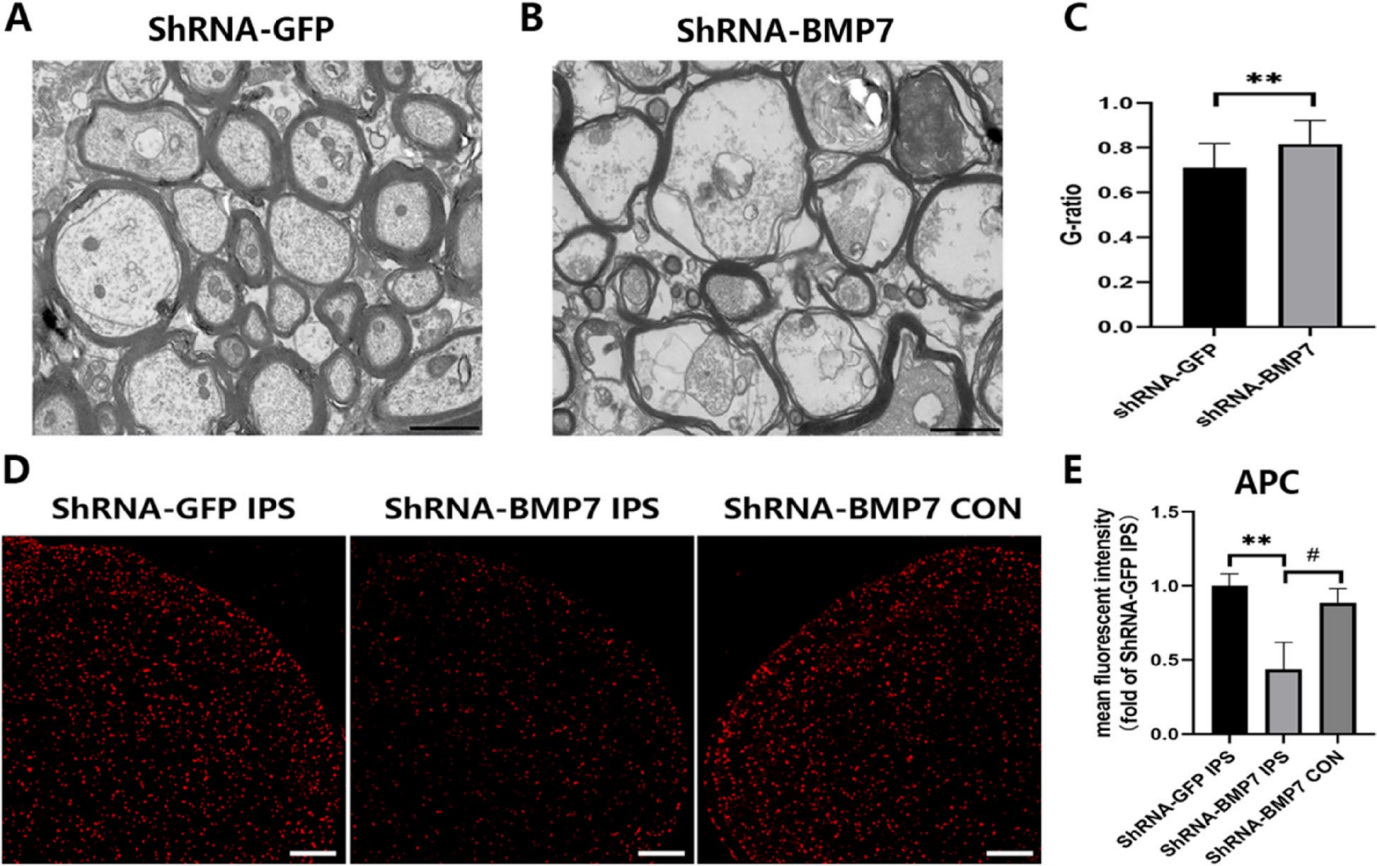
ShRNA-BMP7 caused demyelination and the loss of OLs. **A**, **B** TEM images revealed the myelin profiles in the VC of shRNA-GFP and shRNA-BMP7 groups at day 21 after AAV injection; scale bar = 20 μm. **C** G-ratios of myelin with different diameters which were calculated from (**A**) and (**B**); *n* = 3 per group. **p < 0.01 compared with the shRNA-GFP group. **D** Immunofluorescence staining for APC in the VC of the shRNA-GFP and shRNA-BMP7 groups at day 21 after AAV injection; scale bar = 200 μm. **E** Mean fluorescence intensity of APC was calculated from (**D**); *n* = 3 per group. **p < 0.01 compared with the shRNA-GFP IPS group, ^#^*p* < 0.05 compared with the shRNA-BMP7 CON group. shRNA = short hairpin RNA; IPS = ipsilateral; CON = contralateral; VC = trigeminal spinal subnucleus caudalis

Short hairpin RNAs (shRNAs) can induce stable and heritable sequence-specific gene silencing in mammalian cells. As is normally done with small interfering RNAs (siRNAs), silencing can be provoked by transfecting exogenously synthesized hairpins into cells [16]. Rats in the shRNA-BMP7 group developed mechanical pain hypersensitivity. Compared with the control vector (shRNA-GFP) group, the MWT of the shRNA-BMP7 group was significantly decreased in the ipsilateral ION region from day 14 to 28 after the AAV injection. However, when compared to the baseline (P0), MWT of the shRNA-BMP7 group started to decrease as early as day 7 (Fig. 4A). To confirm the knockdown of BMP7 in the VC, we measured the expression of BMP7 at both the transcription and translation levels at day 21 after the AAV injection. Compared with the shRNA-GFP group, both mRNA (*p* < 0.05) and protein (*p* < 0.05) levels of BMP7 were significantly downregulated by shRNA-BMP7 (Fig. 4B-D). We then tested the expression pattern of CNPase and MBP (markers of OLs) in each group at day 21 after the injection of shRNA-BMP7. The results showed that compared with the shRNA-GFP group, mRNA (*p* < 0.05 or *p* < 0.01) and protein (*p* < 0.05) levels of CNPase and MBP were significantly decreased (Fig. 4E–I). TEM images revealed that compared with the shRNA-GFP group, the myelin sheaths of the shRNA-BMP7 group became thin, loose, and fractured (Fig. 5A, B). To further quantify changes in myelination, g-ratios were calculated from the TEM images. A significant increase in g-ratio was observed in the shRNA-BMP7 group (*p* < 0.01, Fig. 5C). As shown in Fig. 5D, E, immunofluorescence staining revealed that the downregulation of BMP7 in the VC induced a decrease of ipsilateral APC at day 21 after the injection of shRNA-BMP7.

### ShRNA-BMP7 induced OL apoptosis

**Fig. 6:**
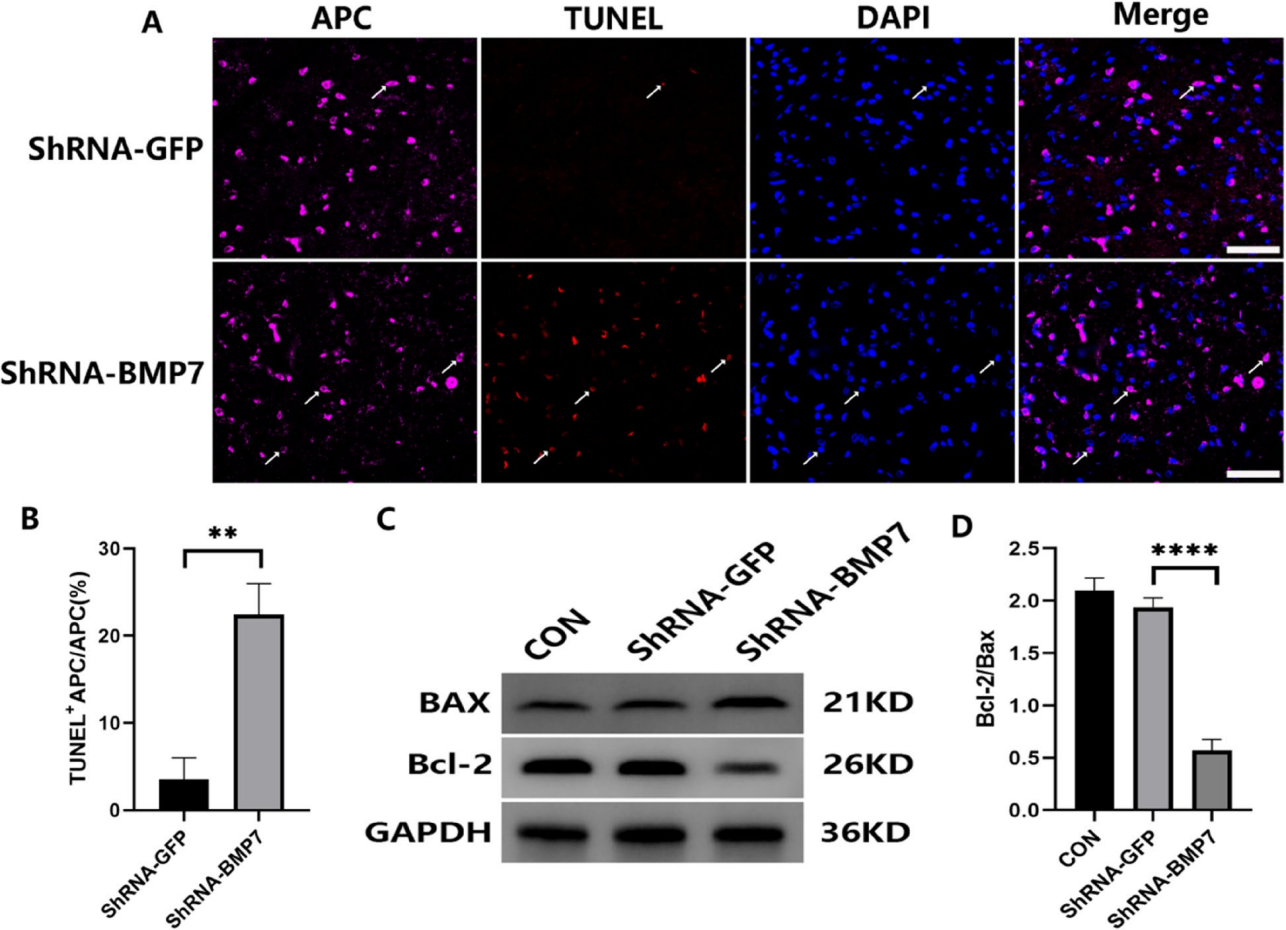
ShRNA-BMP7 induced OL apoptosis in VC. **A** Immunofluorescence of APC (in pink) and TUNEL (in red) in the VC of the shRNA-GFP and shRNA-BMP7 groups at day 21 after the AAV injection. White arrows indicate cells with TUNEL-positive APC staining and DAPI (in blue) labels nucleated cells; scale bar = 50 μm. **B** Quantification of cells with TUNEL-positive APC staining from (**A**); *n* = 3 per group. **C**, **D** Western blotting and the corresponding quantification for Bax and Bcl2 in the VC of CON, shRNA-GFP, and shRNA-BMP7 groups at day 21 after the AAV injection; *n* = 3 per group. Compared with the shRNA-GFP group, **p < 0.01, ****p < 0.0001. shRNA = short hairpin RNA; TUNEL = terminal deoxynucleotidyl transferase-mediated dUTP nick end labeling; VC = trigeminal spinal subnucleus caudalis; AAV = adeno-associated virus

To probe the mechanism of OL loss, the apoptotic events were examined. To explore the possible effect on demyelination induced by downregulating BMP7 expression, the double-immunofluorescence staining for TUNEL and the OL marker APC was performed. We found that downregulation of BMP7 induced OL apoptosis (TUNEL^+^ APC/APC) (**p < 0.01, Fig. 6A, B). To further corroborate this, western blotting of Bax and Bcl-2 was performed. Among a wide range of receptors, Bax is generally associated with apoptosis, while Bcl-2 acts as an antiapoptotic regulator. Therefore, the ratio of Bcl-2/Bax is used to indicate the anti-apoptotic level [17]. The results showed that compared with the shRNA-GFP group, the expression of Bcl2/Bax was significantly reduced in the shRNA-BMP7 group (*p* < 0.0001, Fig. 6C, D).

### AAV-BMP7 relieved mechanical allodynia and demyelination after CCI

**Fig. 7:**
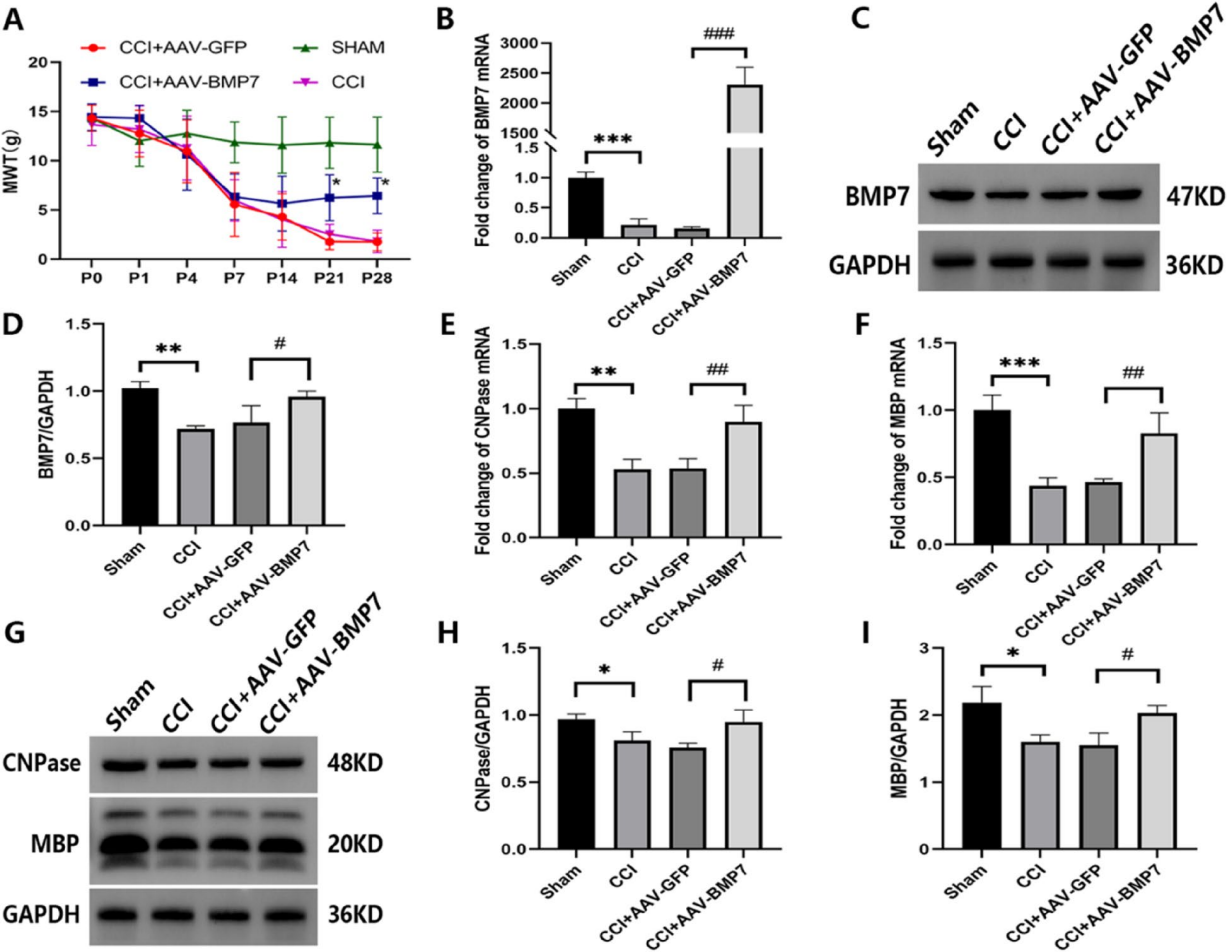
AAV-BMP7 relieved mechanical allodynia and demyelination after CCI. **A** MWT of rats in each group at the indicated time points after CCI; *n* = 6 per group. Compared with the CCI + AAV-GFP group, *p < 0.05. **B**–**D** mRNA and protein levels of BMP7 in the VC of each group at day 21 after the AAV injection; *n* = 3 per group. **E**–**I** mRNA and protein levels of OL markers (CNPase and MBP) in the VC of each group at day 21 after the AAV injection; *n* = 3 per group. mRNA levels in (**B**), (**E**), and (**F**) are presented as a fold change from the Sham group mRNA level. Protein bands depicted in (**C**) and (**G**) have the corresponding optical densities labeled in kiloDaltons (kD). Quantifications of these bands are shown in (**D**), (**H**), and (**I**), normalized by the GAPDH loading control. Compared with the CCI + AAV-GFP group, ^#^*p* < 0.05, ^##^*p* < 0.01, ^###^*p* < 0.001. Compared with the Sham group, *p < 0.05, **p < 0.01, ***p < 0.001. AAV = adeno-associated virus; MWT = mechanical withdrawal threshold; CCI = chronic constriction injury

**Fig. 8:**
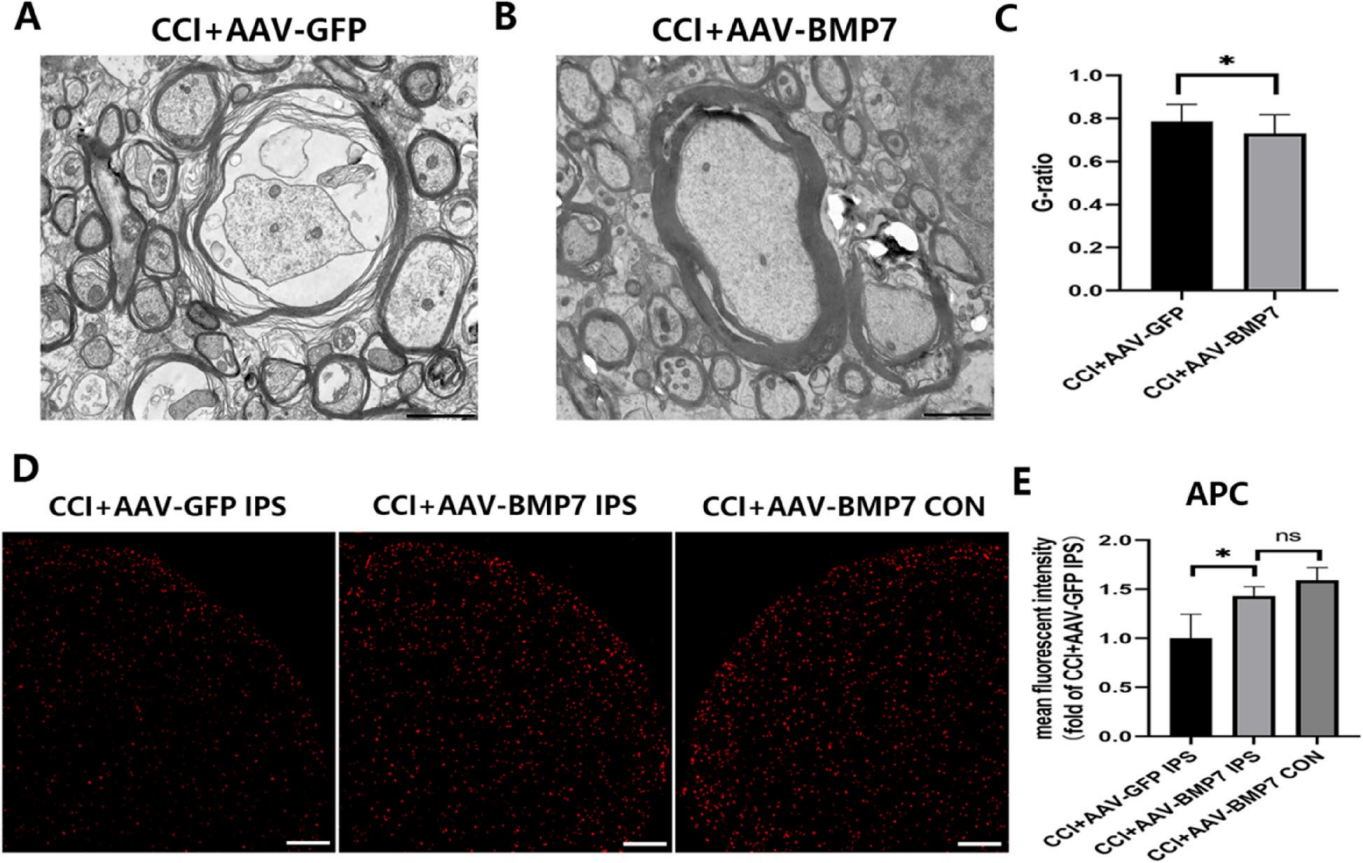
AAV-BMP7 alleviated demyelination and the loss of OLs after CCI. **A**, **B** TEM images show the myelin profiles in the VC of the CCI + AAV-GFP and CCI + AAV-BMP7 groups at day 21 after the AAV injection; scale bar = 20 μm. **C** G-ratios of myelin with different diameters which were calculated from (**A**, **B**); *n* = 3 per group.*p < 0.05 compared with the CCI + AAV-GFP group. **D** Immunofluorescence staining for APC in the VC of CCI + AAV-GFP and CCI + AAV-BMP7 groups at day 21 after the AAV injection; scale bar = 200 μm. **E** Mean fluorescence intensity of APC was calculated from (**D**); *n* = 3 per group.*p < 0.05 compared with the CCI + AAV-GFP IPS group. AAV = adeno-associated virus; OL = oligodendrocyte; CCI = chronic constriction injury; VC = trigeminal spinal subnucleus caudalis

AAV-BMP7 was injected at day 7 after CCI to investigate the role of BMP7 in regulating mechanical allodynia and demyelination. As shown in Fig. 7A, behavioral testing showed that the MWTs were significantly increased after injections of AAV-BMP7 (*p < 0.05). Both mRNA (*p* < 0.001) and protein (*p* < 0.05) levels of BMP7 measured 21 days after the AAV-BMP7 injection were both significantly upregulated in the CCI + AAV-BMP7 group compared to the CCI + AAV-GFP control group (Fig. 7B–D). Therefore, we confirmed that BMP7 was overexpressed in the VC at day 21 after AAV injection. We next examined the expression of CNPase and MBP (markers of OLs) in each group at day 21 after the injection of AAV-BMP7. The results revealed that compared with the AAV-GFP group, mRNA (*p* < 0.01) and protein (*p* < 0.05) levels of CNPase and MBP were significantly increased (Fig. 7E–I). As shown in Fig. 8A, B, compared with the CCI + AAV-GFP group, myelin sheaths of the CCI + AAV-BMP7 group were thicker and denser. Moreover, a significant decrease in g-ratio was observed in the CCI + AAV-BMP7 group (*p* < 0.05, Fig. 8C). Immunofluorescence staining showed that upregulation of BMP7 in the VC induced an increase of ipsilateral APC (OL marker) at day 21 after the injection of AAV-BMP7 (*p* < 0.05, Fig. 8D, E).

### AAV-BMP7 alleviated OL apoptosis after CCI

**Fig. 9:**
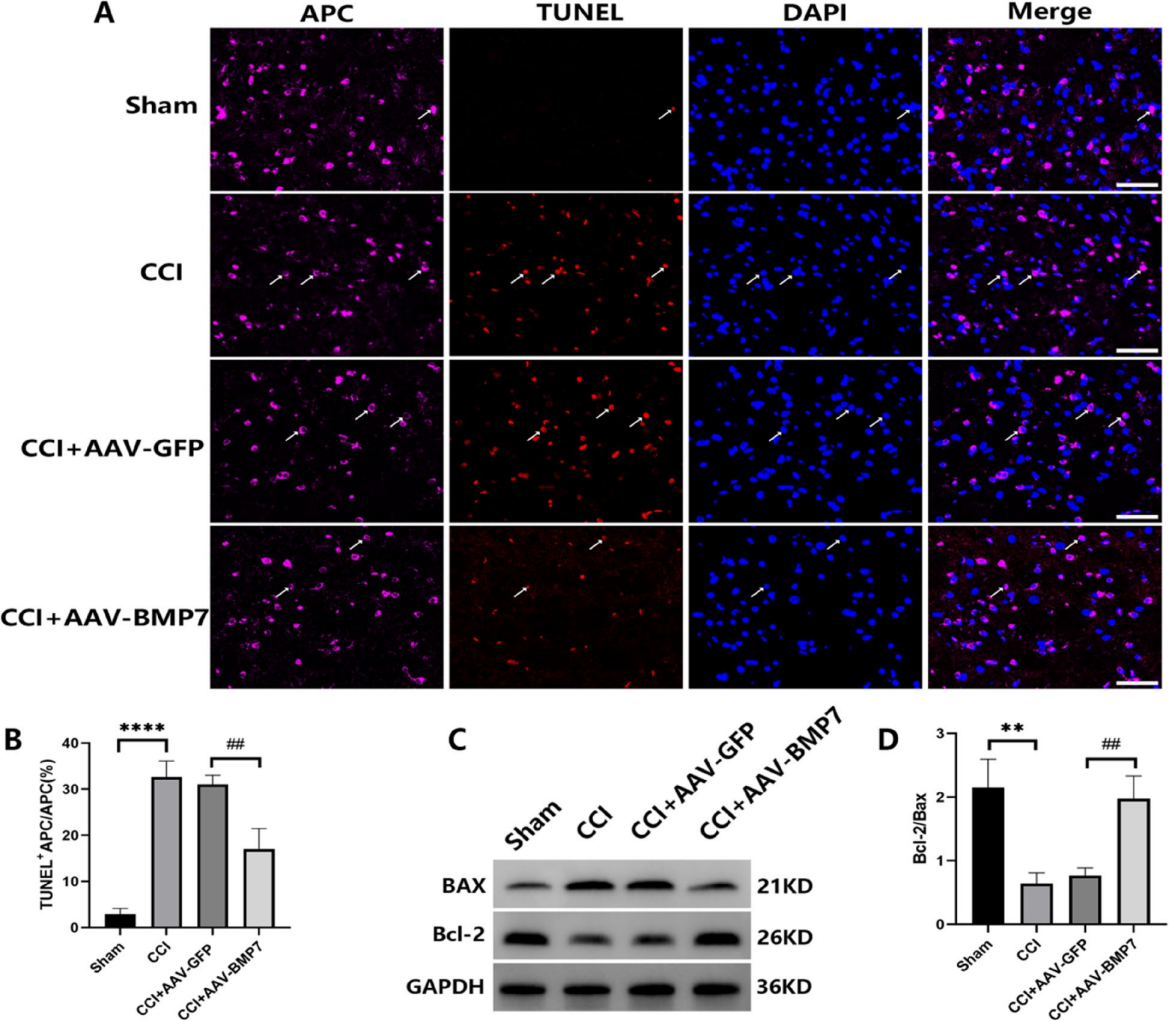
AAV-BMP7 alleviated OL apoptosis in VC. **A** TUNEL (in red) and APC immunofluorescence (in pink) in the VC of each group at day 21 after the AAV injection. White arrows indicate TUNEL-positive APC staining and DAPI (in blue) labels nucleated cells; scale bar = 50 μm. **B** Quantification of cells with TUNEL-positive APC staining from (A); *n* = 3 per group. **C**, **D** Western blotting and the corresponding quantification for Bax and Bcl-2 in the VC of each group at day 21 after the AAV injection; *n* = 3 per group. Compared with the CCI + AAV-GFP group, ^##^*p* < 0.01. Compared with the Sham group, **p < 0.01, ****p < 0.0001. AAV = adeno-associated virus; OL = oligodendrocyte; VC = trigeminal spinal subnucleus caudalis; TUNEL = terminal deoxynucleotidyl transferase-mediated dUTP nick end labeling; CCI = chronic constriction injury

To investigate whether CCI can induce OL apoptosis and if overexpression of BMP7 can alleviate it, we conducted double-immunofluorescence staining for TUNEL and APC, as well as western blotting for Bax and Bcl-2. As shown in Fig. 9A, B, CCI-induced OL apoptosis (*p* < 0.0001) and overexpression of BMP7 alleviated OL apoptosis (*p* < 0.01) in the VC. In addition, CCI caused a decrease in the ratio of Bcl-2/Bax (*p* < 0.01) and upregulation of BMP7 expression induced an increase in the ratio (*p* < 0.01, Fig. 9C, D).

### AAV-BMP7 activated STAT3 and p65

**Fig. 10:**
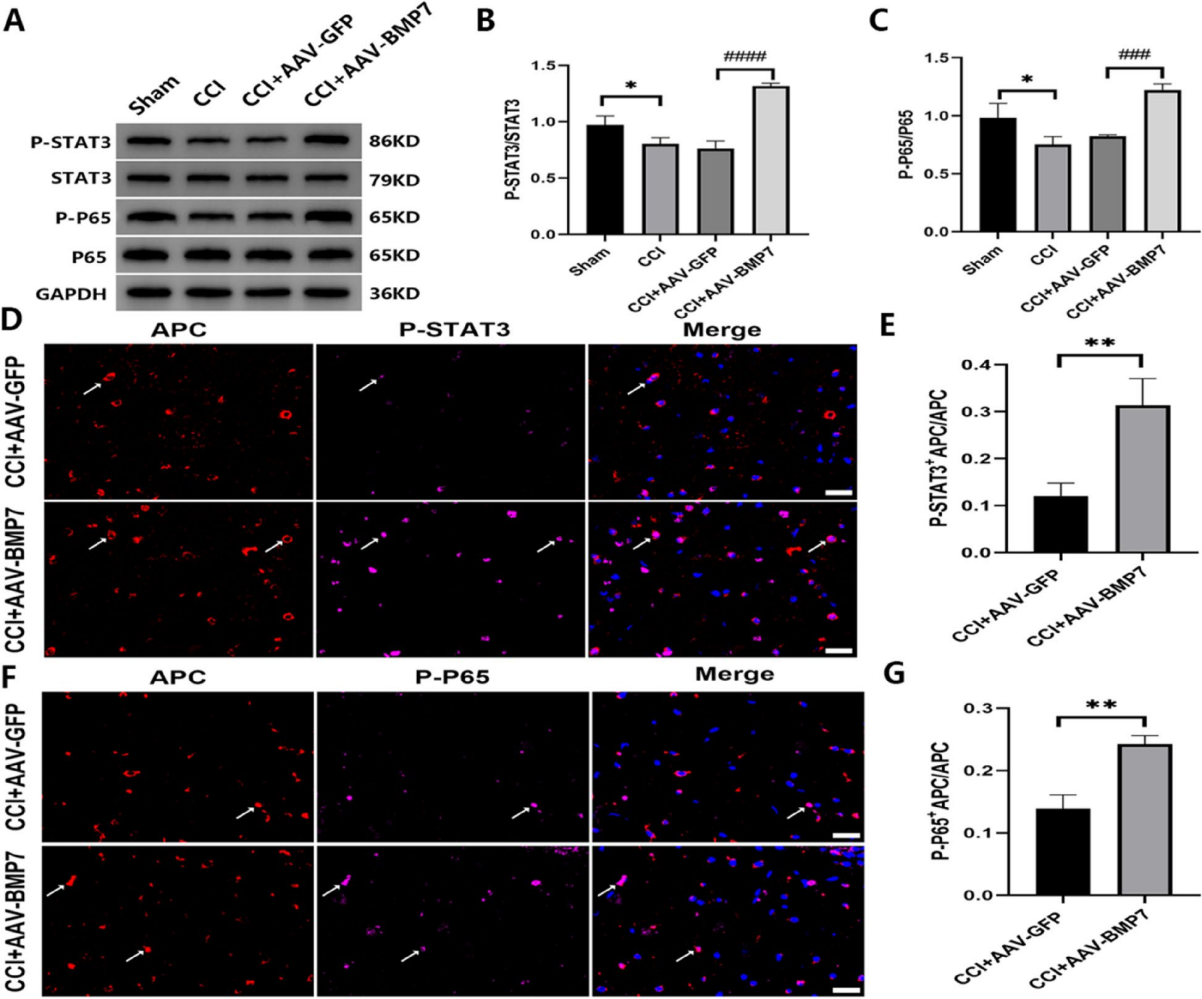
AAV-BMP7 activated STAT3 and p65. **A**–**C** Protein levels of phosphorylated (p)-STAT3, STAT3, p-p65, and p65 in the VC of each group at day 21 after the AAV injection; *n* = 3 per group. Compared with the CCI + AAV-GFP group, ^###^*p* < 0.001, ^####^*p* < 0.0001. Compared with the Sham group, *p < 0.05. **D** The double immunoflurorescent staining of APC (in red) and P-STAT3 (in pink) in the VC of CCI + AAV-GFP and CCI + AAV-BMP7 groups at day 21 after the AAV injection. White arrows indicate P-STAT3-positive APC staining and DAPI (in blue) labels nucleated cells; scale bar = 20 μm. **E** Quantification of cells with P-STAT3-positive APC staining from (**D**); *n* = 3 per group. **F** The double immunoflurorescent staining of APC (in red) and P-P65 (in pink) in the VC of CCI + AAV-GFP and CCI + AAV-BMP7 groups at day 21 after the AAV injection. White arrows indicate P-P65-positive APC staining and DAPI (in blue) labels nucleated cells; scale bar = 20 μm. **G** Quantification of cells with P-P65-positive APC staining from (**F**); *n* = 3 per group. Compared with the CCI + AAV-GFP group, **p < 0.01. AAV = adeno-associated virus; VC = trigeminal spinal subnucleus caudalis; CCI = chronic constriction injury

To further explore the mechanism of how AAV-BMP7 alleviated OL apoptosis after CCI, the expression levels of two classical apoptosis signals, STAT3 and p65, were analyzed. Western blotting results revealed that compared with the sham group, the expressions of phosphorylated-(p-)STAT3 and p-p65 were significantly downregulated (*p* < 0.05) at day 21 after CCI, which was reversed after the AAV-BMP7 injection (*p* < 0.0001 or *p* < 0.001, Fig. 10A-C). While there were occasionally some cells simultaneously marked by P-STAT3 and APC in the CCI + AAV-GFP group, more P-STAT3 was co-located with APC in the CCI + AAV-BMP7 group (Fig. 10D). The proportion of P-STAT3^+^OL in the CCI + AAV-BMP7 group was much higher than that in the CCI + AAV-GFP group (*p* < 0.01, Fig. 10E). Interestingly, P-P65 showed the same trend in OL. AAV-BMP7 induced more P65 phosphorylated in OL (Fig. 10F-G).

## Discussion

CCI-ION stands as a well-established model of trigeminal neuralgia due to its capacity for consistently inducing pain behaviors [13]. More recently, researchers have developed new rodent models, such as FLIT [18] and FRICT-ION [19], for the induction of trigeminal neuralgia. BMPs are secreted proteins that belong to the TGF-β superfamily and regulate proliferation, differentiation, and apoptosis in many different cell types. BMP7 plays a wide role in the development of the central nervous system (CNS). Application of exogenous BMP7 to cultured hippocampal neurons accelerates their dendritic growth [20]. In addition, the lack of BMP7 affects neural development. Ectopic expression of BMP7 in the embryonic midline was shown to increase dentate gyrus neurogenesis, whereas downregulation of local BMP7 signaling reduced embryonic dentate-gyrus neurogenesis [21]. Researchers utilized immunohistochemical methods to demonstrate the expression of BMP7 and BMPR in various cell types of the adult rat spinal cord. Their findings suggest that BMP7 is primarily expressed in oligodendrocytes and neurons, indicating an important role for BMP7 in these cells [22]. In the current study, we observed that the levels of BMP7, CNPase, and APC (markers of OLs) decreased in the VC after CCI. The change in BMP7 expression following CNS injury indicated that the BMP7 signaling pathway may play a role in the development of or recovery from disease [23]. We also found that downregulation of BMP7 in normal rats induced mechanical allodynia, demyelination, and OL loss. These results showed that the presence of endogenous BMP7 is crucial for maintaining the myelin sheath.

To further investigate whether overexpression of BMP7 can alleviate mechanical allodynia and demyelination, AAV-BMP7 as a vector to obtain stable and sustained expression of BMP7 was injected into VC one week after CCI. MWT decreased significantly from day 7 to day 28 after CCI, so we selected rats with decreased MWT for AAV-BMP7 injection at day 7 after CCI. In the current study, we found that BMP7 decreased after CCI, and overexpression of BMP7 relieved mechanical allodynia, demyelination and OL loss after CCI. There is ample evidence in the literature to suggest that BMP7 exerts a neuroprotective effect in various CNS diseases. The local administration of BMP7 following a spinal cord injury has been shown to enhance the number of Nissl bodies in damaged neurons, boost the expression of NF200, and improve the Basso-Beattie-Bresnahan (BBB) scores. The findings provide evidence for the regenerative potential of BMP7 in promoting neuronal recovery after spinal cord injury [24]. BMP7 has shown a protective effect against cerebral ischemia/reperfusion injury in rats, and its neuroprotective effect may be related to reducing oxidative stress and inhibiting neuronal apoptosis [8]. A previous study from our group demonstrated that BMP7 inhibits microglial activation and induces microglial polarization via the STAT3 pathway, thereby attenuating neuroinflammation after spinal cord injury [25]. Similarly, in our current study, BMP7 exhibited a protective effect on rats with CCI by relieving pain behavior, demyelination, and OL loss.

The primary function of oligodendrocytes (OLs) is to create the myelin sheath that surrounds axons, which is crucial for the efficient transmission of action potentials and proper neuronal function [26]. It has been reported that BMP7 can promote the differentiation of OLs [9], inhibit the apoptosis of cultured OLs [11], and reduce the loss of OLs [12] and Schwann cells required for peripheral myelination [27]. But another study showed that BMP7 expression is increased in demyelinated lesions of the spinal cord, which activates astrocytes, increases chondroitin sulphate proteoglycan (CSPG) expression, and then possibly inhibiting remyelination [28]. In the current study, we found that BMP7 was decreased after CCI, and overexpression of BMP7 partly reversed OL loss and alleviated demyelination.

OL apoptosis plays a significant role in demyelination [29–31]. In the current study, TUNEL staining showed that OL apoptosis occurred at day 21 after CCI and downregulation of BMP7, and overexpression of BMP7 alleviated OL apoptosis. To further corroborate this, western blotting of Bcl-2 and Bax was performed, and the ratio of Bcl-2/Bax was used to indicate the anti-apoptotic level [17]. Similarly, we found that the expression of Bcl-2/Bax was decreased after CCI and downregulation of BMP7, and overexpression of BMP7 increased Bcl-2/Bax. We found that CCI and downregulation of BMP7 induced OL apoptosis and overexpression of BMP7 alleviated it, which supports our hypothesis that BMP7-mediated inhibition of OL apoptosis reduces demyelination after CCI. There are many studies suggesting that BMP7 participates in the regulation of cell proliferation and apoptosis in the nervous system. For instance, BMP7 was shown to have a protective effect after cerebral ischemia-reperfusion injury in rats by inhibiting neuronal apoptosis [8]. Additionally, the modulation of the neuroprotective effect of isoflurane on cerebral ischemia-reperfusion injury is associated with reducing neuronal apoptosis by activating BMP7 and p38/MAPK signaling pathways [32]. Finally, BMP7 was found to have neuroprotective effects on amyloid-beta-induced neurotoxicity, and the mechanism of this protection is related to anti-apoptosis and anti-oxidation [33]. As the evidence in the literature focuses on the action of BMP7 on neurons, our study is the first to reveal that BMP7 alleviates TN by reducing OL apoptosis and subsequent demyelination.

STAT3 is closely associated with the wide array of processes in the CNS including cell proliferation, differentiation, inflammation, and survival [34]. There is substantial evidence that activation of the STAT3 signaling pathway significantly reduces apoptosis. One study showed that in delayed recanalization after middle cerebral artery occlusion (MCAO), hepatocyte growth factor (HGF) reduced neuronal apoptosis via a mechanism that included the activation of STAT3 and Bcl2 [35]. Another group reported that sevoflurane post-conditioning reduced apoptosis by increasing the expression of p-JAK2, P-STAT3, and Bcl2 and decreasing that of Bax [36]. An in vitro experiment showed that oxygen-glucose deprivation could trigger apoptosis of mitochondria, while adiponectin (APN) could protect hippocampal HT22 cells from apoptosis by activating the STAT3 signaling pathway [37]. In the current study, we observed that CCI decreased the expression of p-STAT3 and that overexpression of BMP7 increased it. Therefore, we speculate that BMP7 reduces oligodendrocyte apoptosis by activating the STAT3 signaling pathway. Our results are in accordance with previous studies that also suggested that BMP7 can reduce apoptosis via the BMP7/STAT3 pathway [38, 39].

The nuclear factor-κB (NF-κB) is a transcription factor that plays crucial roles in inflammation, immunity, cell proliferation, and apoptosis [40]. Similarly, in our study, phosphorylated p65 (an NF-κB subunit) decreased after CCI and was activated following the upregulation of BMP7. Therefore, p65, like STAT3, could play a role in alleviating apoptosis downstream of BMP7. A previous study indicated that NF-κB prevented TNF-α–induced apoptosis in oligodendrocytes [41]. Other studies have also shown that NF-κB stimulated cell proliferation and blocked apoptosis in different cell types [42, 43]. However, it is also important to acknowledge contradictory results; one research group asserted that inhibiting the TLR4/ NF-κB pathway in human endothelial cells alleviates oxidized, low-density lipoprotein (OxLDL)-induced apoptosis [44]. In addition, a recent study showed that kidney inflammation and apoptosis were attenuated in a mouse model of sepsis-induced acute kidney injury by inhibiting the Src-mediated NF-κB/p65 signaling pathway [45]. Taken together, we conclude that the NF-κB/p65 signaling pathway plays a bidirectional role in apoptosis.

The current study had several limitations. Firstly, only male rats were employed in our experiments due to the perceived challenges associated with evaluating the effects of estrus cycle phases, which change every 4–5 days, on nociception and analgesic responsiveness [46]. Therefore, it is crucial to acknowledge that gender disparities might affect the reliability of our results. Secondly, the BMP7 vector lacked specificity in targeting oligodendrocytes, potentially affecting other cell types like neurons, microglia, and astrocytes. Both microglia [47, 48] and astrocytes [49, 50] can contribute to demyelination by influencing oligodendrocytes. Moreover, the precise relationship between demyelination and neuron loss remains unresolved [51]. In this study, we explored that the overexpression of BMP7 improved demyelination and mitigated the allodynia by enhancing oligodendrocyte survival, but whether it exerted influences through on other cells needs further investigation.

## Conclusion

Our findings revealed that BMP7 can alleviate TN by reducing OL apoptosis and subsequent demyelination in the VC. STAT3 activation and the NF-κB/p65 signaling pathway are potential mechanisms of BMP7 action, but further experiments are needed to provide conclusive evidence of this. Importantly, our study presents clear evidence in support of BMP7 as a possible therapeutic target for the treatment of TN or neuropathic pain.

## Data Availability

The numerous datasets generated in the current study will be made available upon request.

## Abbreviations

BMP7: Bone morphogenetic protein 7
TN: Trigeminal neuralgia
OLs: Oligodendrocytes
ION-CCI: Infraorbital-nerve chronic-constriction injury
shRNA: Short-hairpin RNA
AAV: Adeno-associated virus
TGF-β: Transforming growth factor-β
SCI: Spinal-cord injury
VC: Trigeminal spinal subnucleus caudalis
MWT: Mechanical withdrawal threshold
GAPDH: Glyceraldehyde 3-phosphate dehydrogenase
CNPase: 2’,3’-cyclic nucleotide-3’-phosphodiesterase
MBP: Myelin basic protein
Bax: BCL2-associated x protein
Bcl-2: B-cell lymphoma 2
STAT3: Signal transducer and activator of transcription 3
NF-κB: Nuclear factor-κB
HRP: Horseradish peroxidase
cDNA: Complementary DNA
qPCR: Quantitative real-time polymerase chain reaction
APC: Adenomatous polyposis coli
DAPI: 4´,6-Diamidino-2-phenylindole
TUNEL: Terminal deoxynucleotidyl transferase-mediated dUTP nick end labeling
TEM: Transmission electron microscopy
GFP: Green fluorescent protein
CNS: Central nervous system
